# Role of fatty liver in coronavirus disease 2019 patients’ disease severity and hospitalization length: a case–control study

**DOI:** 10.1186/s40001-021-00590-y

**Published:** 2021-09-26

**Authors:** Arash Ziaee, Ghodsiyeh Azarkar, Masood Ziaee

**Affiliations:** 1 Neuroscience Department, Mashhad University of Medical Sciences, Mashhad, Iran; 2 Infectious Diseases Research Center, Birjand University of Medical Sciences, Birjand, Iran; 3 Infectious Diseases Research Center, Radiology Department, Birjand University of Medical Sciences, Birjand, Iran

**Keywords:** Fatty liver, Coronavirus disease 2019 (COVID-19), Illness severity, Stay length

## Abstract

**Background and purpose:**

Fatty liver is one of the most common pre-existing illnesses; it can cause liver injury, leading to further complications in coronavirus disease 2019 patients. Our goal is to determine if pre-existing fatty liver is more prevalent in hospitalized COVID-19 patients compared to patients admitted before the SARS-CoV-2 pandemic and determine the disease severity among fatty liver patients.

**Experimental approach:**

This retrospective study involves a case and a control group consisting of 1162 patients; the case group contains hospitalized COVID-19 patients with positive PCR tests and available chest CT-scan; the control group contains patients with available chest CT-scan previous to the COVID-19 pandemic. Patients’ data such as liver Hounsfield unit, hospitalization length, number of affected lobes, and total lungs involvement score were extracted and compared between the patients.

**Results:**

The findings indicate that 37.9% of hospitalized COVID-19 patients have a pre-existing fatty liver, which is significantly higher (*P* < 0.001) than the prevalence of pre-existing fatty liver in control group patients (9.02%). In comparison to hospitalized non-fatty liver COVID-19 patients, data from hospitalized COVID-19 patients with fatty liver indicate a longer hospitalization length (6.81 ± 4.76 *P* = 0.02), a higher total lungs involvement score (8.73 ± 5.28 *P* < 0.001), and an increased number of affected lobes (4.42 ± 1.2 *P* < 0.001).

**Conclusion:**

The statistical analysis shows fatty liver is significantly more prevalent among COVID-19 against non-COVID-19 patients, and they develop more severe disease and tend to be hospitalized for more extended periods.

## Introduction

It has been more than 1 year since December 29, 2019, that the first confirmed SARS-CoV-2 case emerged from Wuhan city of China [1], which after a long time, still has so many unknown characteristics. The head of the World Health Organization (WHO) on January 30, 2020, declared the outbreak of COVID-19 to be a public health emergency of international concern and issued a set of temporary recommendations [2], and at the point of writing this study, there have been more than 210 million confirmed cases and more than 4.5 million global deaths. With almost 5 million confirmed cases and more than 100,000 deaths [3], Iran seems to be a good candidate for analyzing virus characteristics. Many researchers started testing different theories through this rough time to identify possible risk factors that affect this disease's severity and mortality rate, including analyzing pre-existing illnesses. These researches include systemic, respiratory, gastrointestinal, and cardiovascular symptoms [4].

According to some studies, liver injury has a notable prevalence in coronavirus disease 2019 (COVID-19) patients, which could be mild (45%), moderate (21%), or severe (6.4%) [5]. Non-alcoholic fatty liver disease (NAFLD) is currently the most common form of chronic liver disease affecting adults and children [6]. These findings become more crucial when we understand that according to one study in China, up to 50% of the people with SARS-CoV-2 had liver dysfunction at some point during their illness [7]. The most significant modifiable risk factors for the poor prognosis from COVID-19 are obesity and metabolic disease [8, 9]. These findings, such as NAFLD, cause the activation of inflammatory pathways [10]. It suggests that NAFLD can play a key role as a risk factor in the severity and prognosis of coronavirus disease 2019 patients. According to a meta-analysis conducted in 2016, the prevalence of NAFLD in Iran is 33.95% [11], and by factoring in lifestyle changes, the prevalence can be estimated to have increased in small amounts through past years. This study gives a more accurate understanding of disease prognosis while having one of the most common pre-existing illnesses.

It should be noted that it is not well understood if COVID-19 makes pre-existing liver disease worse. However, during the COVID-19 pandemic, many infected patients have been treated with antipyretic agents, mainly containing acetaminophen, a drug recognized to cause significant liver damage or induce liver failure [7]. SARS-CoV-2 binds to target cells through angiotensin-converting enzyme II (ACE-2) and uses ACE-2 as the cellular entry receptor [12] ACE2 cellular receptor is highly expressed in human lungs tissues, gastrointestinal tract, and liver [13]. Liver cells can act as a susceptible target for coronavirus disease 2019; however, this mechanism has not been fully confirmed or validated yet [14].

Attention was brought to this topic because of a high number of fatty liver patients while reporting and evaluating COVID-19 patients' lungs involvement scores. Some papers have studied the severity and prognosis of coronavirus disease 2019 using liver enzymes levels such as alkaline phosphatase (ALP), alanine aminotransferase (ALT), aspartate aminotransferase (AST), and viral shedding time [15]. The current study evaluated the severity of COVID-19 patients using different factors such as total lungs involvement score, number of affected lobes, and hospitalization length. The hypothesis was tested to determine if having pre-existing fatty liver can contribute to higher susceptibility, severity, or mortality rate of coronavirus disease 2019.

## Materials and methods

### Ethics

This retrospective study tries to determine if there is a significant correlation between having fatty liver and being more susceptible to COVID-19 and developing more severe disease. The Ethics Committee of the Birjand University of Medical Sciences approved the study (IR.BUMS.REC.1399.187).

### Study design

In this study, 1162 patients were included in the case (*n* = 575) and the control (*n* = 587) groups. Case group patients were selected from Birjand, South Khorasan's Vali-Asr hospital, the main hospital for diagnosing and treating COVID-19 patients. For the control group, data were also selected from the same hospital. A pre-existing fatty liver can be determined by measuring the patient's liver Hounsfield unit (HU) based on their imaging data, which reports radiodensity on a quantitative scale. Hounsfield units are mainly used to report the fat content of the liver and diagnose pre-existing fatty liver. According to references and protocols, patients with a HU of 40 or below are considered fatty liver patients [16]. In this study, patients with a borderline score of 40 were evaluated once more to reduce the bias and develop more accurate results.

Chest CT-scan images were taken by Siemens SOMATOM Emotion 16 Slice CT-scan machine.

The data were extracted using the hospital picture archiving and communication system (PACS).

The severity of the disease was evaluated using three factors:

1. Days of hospitalization,
2. The number of affected lobes ranges from 0 to 5,
3. Total lungs involvement (chest severity) ranges from 0 to 20.

Using Table 1, guideline involvement scores were calculated separately for upper, middle, and lower lobes and individually for right and left lung. The sum of each lobe’s scores gives total lungs involvement. The guideline is on par with lungs involvement measurement protocols set by the Iran health department and is the primary method used for evaluating lungs severity in Iran.

**Table 1:** Lobes involvement score

Percentage of lobe involvement	Score
< 25	1
25–49	2
50–75	3
> 75	4

### The case group

The case group consisted of hospitalized COVID-19 patients, all of whom had positive polymerase chain reaction (PCR) tests and a chest CT-scan. Each hospitalized patient underwent a CT-scan on the first day of admission. Patients were chosen from March 2020 through November 2020. The evaluated and collected data include liver HU, sex, age, admission date, total lungs involvement score, the number of affected lobes, and the hospitalization length.

The data were analyzed to measure the prevalence of fatty liver in hospitalized COVID-19 patients. At first, the prevalence of fatty liver was analyzed based on sex, age, and admission date. The case group was then divided into two groups based on having pre-existing fatty liver. Total lungs involvement score, the number of affected lobes, the hospitalization length, and mortality rate were compared between these two groups based on different factors, such as sex, age, and month of the year.

### The control group

The control group consists of all patients who have had a chest CT-scan a year prior to the COVID-19 outbreak from March 2019 through the end of November 2019. Chest CT-scan could have been performed for various reasons, but it is not performed for any reason relating to COVID-19 disease and data are not available to know if patients were hospitalized. The collected data include liver HU, sex, age, and admission date. Fatty liver prevalence was then measured and analyzed based on sex, and age, and date. The control group was then divided into two groups based on having pre-existing fatty liver. Sex distribution was then compared.

For the primary analysis, the prevalence of fatty liver was compared between hospitalized COVID-19 patients (case group) and non-COVID-19 patients (control group).

### Inclusion criteria

All patients admitted from March 2020 through November 2020 were included in the case group if they met the criteria that included COVID-19 confirmation using PCR test, admission and hospitalization, available chest spiral CT-scan, and access to their imaging data for liver HU measurement.

All patients from March 2019 through the end of November 2019 were included in the control group if they met inclusion criteria that included available chest spiral CT-scan, administration before December 2019, and available access to their imaging data for measuring liver HU.

### The exclusion criteria

Patients aged under 18 years were excluded from participation from both the case and the control groups. If a hospitalized COVID-19 patient had two or more chest CT-scan, only the first imaging data were used to evaluate fatty liver scores.

### Statistical analysis

In order to control and balance heterogenicity between two groups, exclusion criteria were sought to be small so that control and case group patients would have the same heterogenicity. For statistical analysis, patients were grouped into six age groups: under 30, 30 to 40, 40 to 50, 50 to 60, 60 to 70, and upper than 70.

Categorical variables were compared using the Chi-squared test, and between-group comparisons were assessed using unpaired *t*-tests. A scatterplot matrix was used to visualize and give a descriptive analysis of bivariate relationships between combinations of variables. Quantitative data were presented as mean, standard deviation (SD), or median. A *P*-value of 0.05 and below was considered statistically significant. Statistical Package for the Social Sciences (SPSS) version 22 software was employed for data analysis. Statistical analysis was performed only on patients with a complete set of data; if the patient's data were incomplete, they were excluded from the analysis.

## Results

### Fatty liver distribution

The study consists of 1162 patients, and it includes a case group of 575 hospitalized patients with confirmed COVID-19 infection and a control group of 587 patients with chest CT scans in 2019. No patient had missing data. The prevalence of pre-existing fatty liver among hospitalized COVID-19 patients (case group) was significantly higher than the control group patients (37.9% vs. 9.02% *P* < 0.001).

When the case group is divided based on having pre-existing fatty liver, the percentage of male patients is significantly higher in the group with pre-existing fatty liver in comparison to the group without fatty liver (60.8% vs. 50.7%, *P* = 0.02). However, there is no significant difference in the male gender’s distribution among non-COVID-19 patients if divided by the pre-existing fatty liver (42.3% vs. 44.8%, *P* = 0.77). The distribution of fatty liver in the case group was significantly more concentrated in the 51–60 years age group (*P* = 0.01).

### COVID-19 severity and mortality

The severity of the disease was compared among COVID-19 patients divided into two groups based on having pre-existing fatty liver. The virus seems to affect more lobes (4.42 ± 1.2 *P* < 0.001), leading to a higher total lungs involvement score (8.73 ± 5.28 *P* < 0.001) among COVID-19 patients who had pre-existing fatty liver. COVID-19 patients with fatty liver are hospitalized for more extended periods (6.81 ± 4.76, *P* = 0.02).

Multivariable analysis of three previous factors showed a total *P*-value of < 0.001. Interestingly, while the results suggest that COVID-19 patients with fatty liver develop a more severe form of COVID-19, the findings do not show a significantly higher risk of mortality for patients with pre-existing fatty liver (11.5% vs. 10.1%, *P* = 0.58). However, deceased patients' lungs involvement scores (11.9 ± 6.25, *P* < 0.001) and the number of affected lobes (4.52 ± 1.16, *P* = 0.005) are significantly higher than survived patients. The elderly patient's mortality rate was noticeably higher among the case group patients (74.56% ± 11.99, *P* < 0.001).

According to our results, there is not a significant difference between the male and female patient's disease severity and mortality. Male patients’ total score of lungs involvement (7.4 ± 5.05 *P* = 0.17), number of their affected lobes (4.01 ± 1.53 *P* = 0.13), and their hospitalization length (6.39 ± 4.9 vs. 6 ± 4, *P* = 0.32) are not significantly higher than female patients. Men are also not at a significantly higher risk of COVID-19 mortality (60.7%, *P* = 0.31).

Our study shows the severity of the disease is increased in months of autumn in Iran (September–November) with a higher total score of lungs involvement (10.36 ± 4.94, *P* < 0.001) and a more significant number of affected lobes (4.79 ± 0.70, *P* < 0.001). In contrast, hospitalization lengths are significantly longer in the first month of spring in Iran, which is from March through April (8.9 ± 6.61, *P* = 0.005).

### Factors correlation

**Fig. 1:**
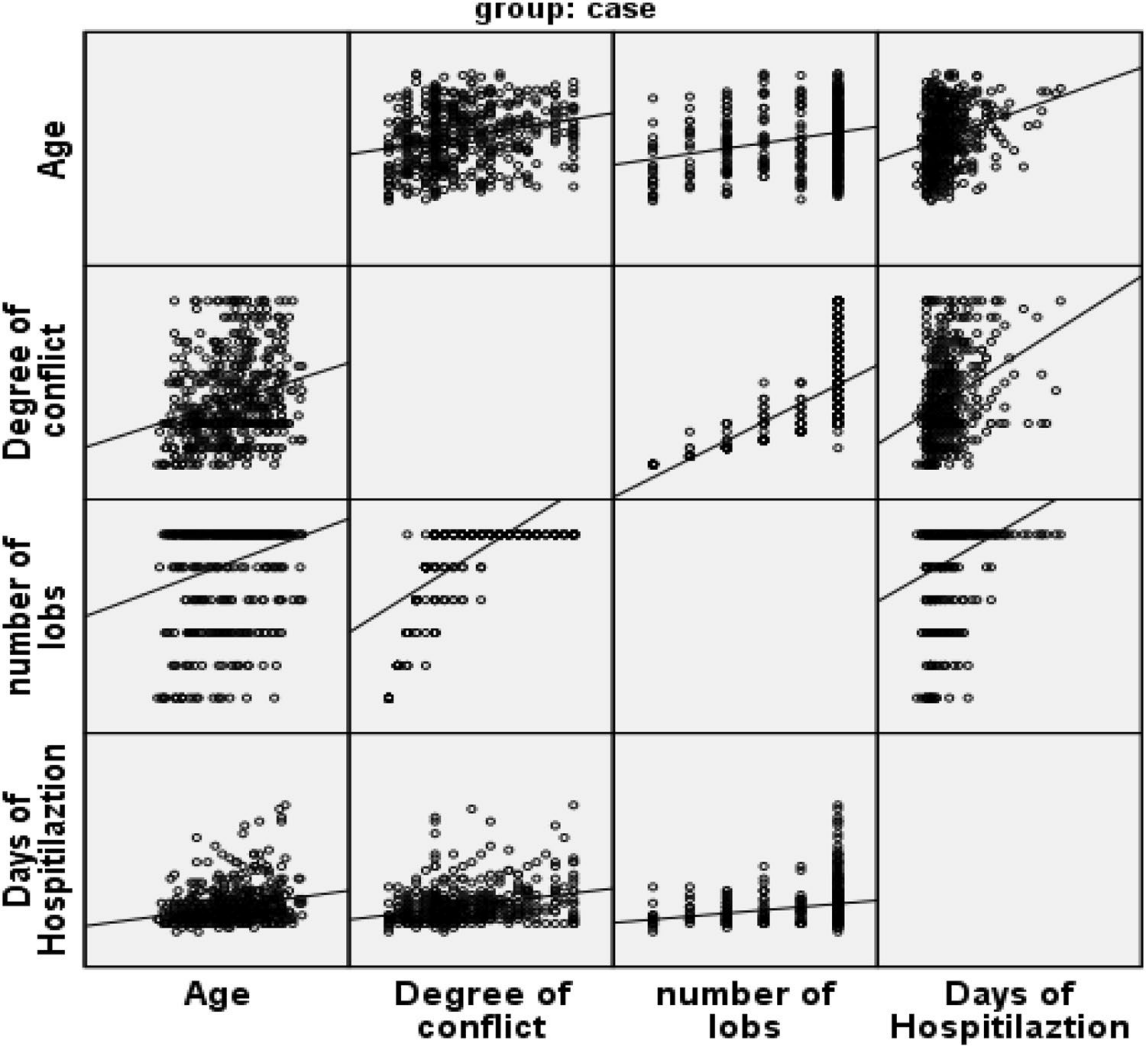
Scatterplot matrix analysis shows the correlation of lungs involvement score, number of affected lobes, and hospitalization length, and age

Analysis of bivariate correlations between combinations of variables (Fig. 1) that includes lungs involvement, number of affected lobes, hospitalization length, and age shows the following results: (1) total lungs involvement score (*r* = 0.24, *P* < 0.001); (2) number of affected lobes (*r* = 0.27, *P* < 0.001), and (3) hospitalization length (*r* = 0.24, *P* < 0.01). The analysis shows elderly patients are more susceptible to be infected with COVID-19 and develop more severe disease with a higher total lungs involvement score and a more extended hospitalization period.

### Sex distribution

The percentage of male patients in the case group is significantly higher than the control group (54.5% vs. 44.5%, *P* 0.001). In the control group, when divided based on having pre-existing fatty liver, males have higher average age than females in the group of patients without fatty liver (59.15 ± 18.91, *P* = 0.03) (Table 2).

**Table 2:** Comparison of sex distribution in the control group, analyzed by average age, average Hounsfield unit

The control group total number of patients = 587		Non-COVID-19 patients with fatty liver	Non-COVID-19 patients without fatty liver
The average age	Male	55.14 ± 18.41	59.15 ± 18.91
	Female	56.43 ± 15.20	55.71 ± 17.45
	*P-*value	0.78	0.03
The average liver Hounsfield unit	Male	33.18 ± 7.90	56.21 ± 7.52
	Female	35.37 ± 5.22	57.63 ± 7.99
	*P-*value	0.23	0.04

In the case group, when divided based on having pre-existing fatty liver, females have higher average age than males among COVID-19 patients with fatty liver (59.29 ± 13.45, *P* = 0.03); while among COVID-19 patients without fatty liver, the average female age is not significantly higher than males. The number of affected lobes (4.61 ± 1.09, *P* = 0.05) and total score of lungs involvement (9.68 ± 5.70, *P* = 0.04) are noticeably higher among female COVID-19 patients with fatty liver. There is no significant difference between each gender's mortality rate among COVID-19 patients with fatty liver. In contrast, the male mortality rate is higher in COVID-19 patients without fatty liver (69.4%, *P* = 0.02) (Table 3).

**Table 3:** Comparison of sex distribution in the case group, analyzed by average age, average liver Hounsfield unit, average number of involved lobes, average score of lungs involvement, and mortality

The case group total number of patients = 575		COVID-19 patients with fatty liver	COVID-19 patients without fatty liver
The average age	Male	54.53 ± 18.85	58.31 ± 20.41
	Female	59.29 ± 13.45	57.77 ± 17.67
	*P-*value	0.03	0.79
The average Hounsfield unit	Male	32.64 ± 7.89	50.49 ± 6.03
	Female	30.99 ± 8.88	51.30 ± 6.72
	*P-*value	0.16	0.23
The average days of hospitalization	Male	6.83 ± 4.98	6.07 ± 4.87
	Female	6.77 ± 4.44	5.63 ± 3.74
	*P-*value	0.93	0.36
The average number of affected lobes	Male	4.29 ± 1.25	3.81 ± 1.67
	Female	4.61 ± 1.09	4.01 ± 1.55
	*P-*value	0.05	0.25
The average total score of lungs involvement	Male	8.11 ± 4.91	6.90 ± 5.11
	Female	9.68 ± 5.70	7.20 ± 5.22
	*P*-value	0.04	0.57
Deceased Total: 62	Male	48%	69.4%
	Female	52%	30.6%
	*P-*value	0.16	0.02

## Discussion

Our results showed that fatty liver is significantly more prevalent in COVID-19 patients, which is on par with other studies stating that fatty liver has a higher percentage among COVID-19 patients in comparison with non-COVID-19 patients [10, 15]. The fatty liver prevalence among hospitalized COVID-19 patients is higher than the calculated prevalence of NAFLD in Iran from 2016 (37.84% vs. 33.95%). Some studies' findings state that increased liver fibrosis in NAFLD might affect COVID-19 outcome [17]. Our results are also supported by Bramante et al.’s study, which indicates fatty liver patients have a much higher risk of COVID-19 hospitalization. That study suggested metabolic syndrome and NAFLD/NASH available treatments significantly mitigated risks of COVID-19, those with home metformin glucagon-like-peptide 1 receptor agonist (GLP-1 RA) use have a non-significantly reduced odds of hospitalization [9].

Our study demonstrates that COVID-19 patients, who suffer from fatty liver, have to be hospitalized for more extended periods, which is confirmed by the study of Dong Ji and colleagues [15]. Data analysis also shows that patients with fatty liver experience more severe symptoms during the disease. The number of involved lobes and total lungs involvement scores are higher in patients with pre-existing fatty liver, which can be attributed to the findings of the extended period of hospitalization data. A higher risk of disease progression is also suggested by another study that evaluated the disease severity by different factors [15]. Another study also confirms our findings that fatty liver patients experience a more severe form of the disease [18].

In addition, the results suggest that social awareness should be promoted regarding the negative impact of metabolic diseases such as pre-existing fatty livers on patients with COVID-19 and that health policymakers should promote the use of preventive measures to control obesity and fatty liver.

With increased disease severity, the mortality rate of the coronavirus disease 2019 was expected to be noticeably higher among fatty liver patients. However, from the data analysis results of the current study, it could not be concluded that fatty liver is linked to a higher COVID-19 mortality rate. This is in contrast with another study that concludes liver injury is strongly associated with the COVID-19 mortality risk [9, 18].

According to our findings, the severity of COVID-19 is increased in Iran's autumn months, which is from September through October. It can be confirmed by other studies that suggest the emergence of virus mutations could have made the COVID-19 virus more transmissible and infectious [19]. COVID-19 hospitalization length was not linked to autumn; however, it was longer at the beginning of the COVID-19 pandemic. It can be speculated that patients used to be hospitalized for more extended periods because of not fully known treatment and hospitalization protocols. We suggest COVID-19 had a higher disease severity in the autumn; however, it should also be noted that the number of patients drastically increased during the autumn. Therefore, hospitals could only admit patients with more severe symptoms. A newly conducted study also suggests that the increase in the number of COVID-19 patients and severity could be related to the decrease in the individuals' vitamin D levels in the autumn and winter seasons [20]. Previous studies give a clear understanding that there is an essential and direct role for vitamin D in modulating liver inflammation and fibrogenesis [21, 22]. Other studies show a clear correlation between COVID-19 and vitamin D deficiency [23, 24], which indicates that treatment of fatty liver patients' vitamin D deficiency can reduce the chance of liver injury [24] and ultimately decrease coronavirus disease 2019 severity and mortality [9, 25].

According to our findings and similar studies, the percentage of male patients is significantly higher than women in COVID-19 [26]. However, our findings cannot validate the theory that male patients are also more prone to more severe forms of the disease, which is contrary to the study of Kuno et al. [27]. Scatterplot matrix data analysis showed that older adults are more susceptible to developing a more severe form of the disease and have to be hospitalized for more extended periods. Their total score of lungs involvement is significantly higher, which is validated by previous studies. It can also be attributed to pre-existing illnesses [28]. The elderly male mortality rate is higher than expected, and it is validated by other studies [29].

### Limitations

Deceased patients' data could only be collected from June through August and October through November of 2020. Hence the number of deceased patients could not be compared between different months of the year. The deceased patients' data were only used to compare mortality between the COVID-19 patients grouped based on pre-existing fatty liver and determine if one gender has a higher risk of mortality. There was no access to each patient's past medical history. Thus, patients could not be accurately categorized into non-alcoholic fatty liver disease patients. Therefore, the term “fatty liver patients” was used in this study. The lack of diagnosis data for control group patients limited us from removing patients with the diseases that could cause fatty liver. However, based on the date of the CT-scan, it was assumed that the chest CT-scan was not related to COVID-19.

## Conclusion

The study concludes that fatty liver can play a crucial role in susceptibility to being infected with SARS-CoV-2 and the severity of COVID-19 patients. The prevalence of fatty liver in COVID-19 patients is significantly higher than non-COVID-19 patients. COVID-19 patients with pre-existing fatty liver are hospitalized for more extended periods and have a higher total lungs involvement score.

The results also further confirm findings from previous studies that male and elderly patients are more prone to coronavirus disease 2019 infection. In contrast to other studies, our findings show that male and elderly patients are not at a higher risk of disease severity and mortality.

Treatment for obesity and pre-existing metabolic disease should be a priority while knowing this significantly higher risk. Therefore, prospective studies are necessary to determine the exact cause and effect correlation between SARS-CoV-2 and fatty liver.

## Data Availability

All of the authors of “Role of Fatty Liver in Coronavirus Disease 2019 Patients' Disease Severity and Hospitalization Length: A Case–Control Study” confirm that the data supporting the findings of this study are available within the article and its supplementary materials. A complete set of data will be provided upon request to the reviewers if it is necessary, but due to the fact that COVID-19 is still not a well-known disease, there are confidentiality concerns around COVID-19 patients’ data; therefore, the data cannot openly be deposited in repositories and supporting data cannot be made openly available.

## Abbreviations

HU: Hounsfield unit
COVID-19: Coronavirus disease 2019
WHO: World Health Organization
NAFLD: Non-alcoholic fatty liver disease
ACE-2: Angiotensin-converting enzyme II
ALP: Alkaline phosphatase
ALT: Alanine aminotransferase
AST: Aspartate aminotransferase
PACS: Picture Archiving and Communication System
PCR: Polymerase chain reaction
NASH: Non-alcoholic steatohepatitis
GLP-1 RA: Glucagon-like-peptide 1 receptor agonist
